# Relationship Dynamics Underlying Cancer Overtreatment in Advanced Cancer Patients From an Oncologist Point of View

**DOI:** 10.3389/fpsyg.2021.754432

**Published:** 2021-11-18

**Authors:** Fabrizio Artioli, Giorgia Razzini, Dania Barbieri, Cesare Secchi

**Affiliations:** ^1^Unit of Medical Oncology, Civil Hospital “B. Ramazzini”, Azienda USL Modena, Carpi, Italy; ^2^Unit of Psychology, Civil Hospital “B. Ramazzini”, Azienda USL Modena, Carpi, Italy; ^3^International Psychoanalytical Association, Reggio Emilia, Italy

**Keywords:** cancer, overtreatment, oncologist, patient, relationship, hope

## Introduction

The burden of cancer in Europe is estimated to have risen to 2.7 million new cases and 1.3 million deaths in 2020 (European Network of Cancer Registries, 2020). When comparing data over different time periods, it can be said that about half of cancer patients die because of their disease.

To prolong life, as well as to relieve symptoms, patients with advanced or relapsed cancer are over treated with antineoplastic agents before they die.

Despite the crucial contribution of integrating early palliative care in cancer management (Temel et al., 2010; Bandieri et al., 2020), no definitive change in the overtreatment of cancer patients with advanced disease, particularly those at the end of life, has been seen yet (Martoni et al., 2017; Zhang et al., 2021). Hospitalization in advanced-stage disease, given its poor prognosis, can itself be considered a form of overtreatment (El-Jawahri et al., 2020).

From 2003 to 2010, the use of chemotherapy increased by 67% in the U.K., which led to an excessive “pharmaceuticalization” in oncology. A similar phenomenon, albeit to a lesser extent, was seen in other Western countries (Davis, 2015). Nevertheless, chemotherapy in cancer patients with advanced disease is often ineffective (Rochigneux et al., 2017) and aggressive (Pacetti et al., 2015).

With the advent of molecular targeted therapy and immunotherapy, the drugs available to oncologists over the last 20 years have increased by 70%.

No one denies the improvement, even considerable improvement, provided by these new therapies to the survival of patients with metastatic cancer. It is equally true, however, that many of these treatments do not meet the expectations of patients in terms of prognosis, or even sometimes do not correspond to the results of randomized controlled clinical trials (Fojo and Parkinson, 2010).

Given that oncologists frequently avail themselves of anticancer drugs, the expectations of patients concerning their life expectancy have likewise increased. However, prescribing ineffective cancer treatment can be considered a substitute for a relationship that becomes more and more difficult as the disease worsens.

The aim of this commentary is to reflect on this theme, with particular reference to oncologist-patient relationship dynamics in facing end-of-life communication.

## Cancer Overtreatment as Therapeutic Illusion

When the cancer of a patient becomes advanced, the oncologist-patient relationship changes. While aware of the fact that the only outcome possible is the death of the patient, oncologists are often reluctant to communicate poor prognosis.

Faced with the death of a patient, the most convenient option available to the oncologist is often to prescribe further anticancer therapy, as if the metastatic threshold had not been crossed, with the implied objective being to maintain the *status quo* of living with cancer (or even to achieve complete recovery). Notably, cancer patients with advanced disease claim they do not know that their prognosis is poor, or that the treatment they are undergoing is only palliative (Weeks et al., 2012). The patient is deeply reassured, and the oncologist feels as if the disease can still be controlled. Thus, they complicitly deny death, or even the worsening of the disease; the void created by the unsaid is filled and exorcized by a multiplication of medical interventions (treatments, medical visits, diagnostic tests).

When informed that their clinical situation has worsened, patients with cancer often turn to another specialist for a second opinion. These patients are looking for a more complete explanation regarding how serious their disease really is, for treatments that are potentially more effective, or even only to be reassured that their oncologist is managing their case appropriately (Hillen et al., 2017). The oncologist experiences the patient's search for a second opinion as a defeat, which is, at times, accompanied by the more or less explicit fear that another oncologist will not confirm the appropriateness of cancer management so far. A second opinion, as the patients' right, should lead to a discussion of the case among colleagues and be shared with the attending oncologist (Payne et al., 2014), but it often leads to overtreatment (Philip et al., 2010).

Cancer overtreatment cultivates the illusion that there are endless therapeutic solutions, which implies the omnipotence of medicine and immortality of the patient. A therapeutic pseudo-alliance is formed, which is presented as temporarily adaptive but definitely dysfunctional for the patient-oncologist relationship.

When the disease persists, and even more so when it worsens irreversibly, the mind of the patient, subject to unfamiliar emotional pressure, may cling to miraculous fantasies. As Freud reminds us (Freud, 1918), “*At bottom, no one believes in his own death, or, to put it another way, in the unconscious every one of us is convinced of his own immortality*”.

In the initial phase of facing their disease, patients, while expecting to recover, still fantasize about the progression of their cancer and of impending death. Subsequently, when their cancer responds to treatment, they hope to avoid any recurrence, sometimes resorting to thoughts and behaviors that give them a sense of regaining that initially lost control.

Thus, at least right then and there, the patient with advanced cancer accepts the proposal of the oncologist to continue with further cancer therapies; this allows both to avoid facing the end-of-life experience.

## Open and Honest Communication as a Hope-Giving Process to Reduce Overtreatment

We strongly believe that when all effective cancer therapies have been exhausted, the oncologist must inform the patient of the prognosis openly and honestly.

This moment can be dramatic for the patient, who must not only give up any idea of surviving but also risks feeling abandoned, no longer counted among the curable, resulting in death anxiety in the patient (Gonen et al., 2012).

In his article, Josébustamante (2001) observes that subjects deal with critical situations according to their personality and their way of hoping. Strongly reasserting the principle of residual quality of life, Bustamante emphasizes the need to understand the patient as a whole person, with all the prerogatives of a symbolic animal (conscience, emotions, inner world, belief system, need to love and be loved).

Should the prognosis worsen, the process of hope deepens, and the oncologist should change accordingly to accompany the patient in this delicate phase of the end of life (Carrieri et al., 2020).

Facing death, a new type of hope emerges, fully rooted in the historical and personal reality of each individual, including, but not limited to, the hope that pain will disappear, the hope of receiving the visit of a loved one, the hope of life after death, and so on. In light of these reflections, hope would ascend to an existential category of great importance at the end of life (Eliott and Olver, 2007).

Being able to think of a life after, and without, oneself in the here and now is a psychic process of extraordinary significance and awareness; it is not just one generic expedient among many, but a realistic point of arrival. Accompanied and supported by the oncologist, the patient's processing can generate further hope and comfort, even to the point of achieving self-reconciliation. Thus, an open and honest communication between the oncologist and the patient can itself be considered as a hope-giving process.

The pain involved in direct communication naturally concerns oncologists as well, who must admit that they can no longer keep their patient alive for long; they must find within themselves the courage and willingness to navigate end-of-life care with empathy and lucidness. Nevertheless, since this process can also activate anguish, guilt, and suffering, oncologists must forestall this risk with an attitude that is as flexible and sensitive as possible (Draper et al., 2019).

In this view, a psycho-oncologist helps to support and integrate the oncologist by assisting in understanding the psychological dimension and experience of the patient related to the disease. Moreover, both the patient and the oncologist can share their fears and fatigue in a safe space for reflection and emotional listening (Teo et al., 2019).

We are aware that psycho-oncologists and palliative care teams are essential to supporting patients with terminal cancer; however, delegating end-of-life care exclusively to them entails the interruption of the oncologist-patient relationship (Carrieri et al., 2020).

Oncologists often lack the training to communicate effectively, despite several studies having emphasized that successful communication promotes the wellbeing of the patient and of the health care staff (Levit et al., 2013). It would, therefore, be useful to promote training in medical schools as well as professional educational programs for cancer teams such as Balint groups (Bar-Sela et al., 2012) and multi-professional team discussions of cases, in order to improve the communication between the patient and the oncologist throughout the course of disease. Although there is no one-size-fits-all plan for a specific clinical scenario, the main purpose is to ensure that oncologists are well equipped to provide high-quality end-of-life communication to their patients, while at the same time recognizing and understanding their own feelings and anxieties about dying and death (Draper et al., 2019).

Therefore, we strongly encourage that oncologist communication moves toward greater transparency on cancer prognosis, sharing treatment decision-making with the patient, reducing potential cancer overtreatment, and improving end-of-life care.

## Author Contributions

All authors listed have made a substantial, direct, and intellectual contribution to the work and approved it for publication.

## Conflict of Interest

The authors declare that the research was conducted in the absence of any commercial or financial relationships that could be construed as a potential conflict of interest.

## Publisher's Note

All claims expressed in this article are solely those of the authors and do not necessarily represent those of their affiliated organizations, or those of the publisher, the editors and the reviewers. Any product that may be evaluated in this article, or claim that may be made by its manufacturer, is not guaranteed or endorsed by the publisher.
